# Is Sunlight Exposure Enough to Avoid Wintertime Vitamin D Deficiency in United Kingdom Population Groups?

**DOI:** 10.3390/ijerph15081624

**Published:** 2018-08-01

**Authors:** Richard Kift, Lesley E. Rhodes, Mark D. Farrar, Ann R. Webb

**Affiliations:** 1 School of Earth and Environmental Science, Faculty of Science and Engineering, University of Manchester, Manchester M13 9PL, UK; ann.webb@manchester.ac.uk; 2 Division of Musculoskeletal and Dermatological Sciences, School of Biological Sciences, Faculty of Biology, Medicine and Health, University of Manchester, and Dermatology Centre, Salford Royal NHS Foundation Trust, Manchester Academic Health Science Centre, Manchester M6 8HD, UK; lesley.e.rhodes@manchester.ac.uk (L.E.R.); mark.farrar@manchester.ac.uk (M.D.F.)

**Keywords:** vitamin D, ultraviolet radiation, climatology, white Caucasian, South Asian, adolescent, photodermatoses

## Abstract

Solar ultraviolet radiation (UVR) is required for cutaneous vitamin D synthesis, and experimental studies have indicated the levels of sun exposure required to avoid a vitamin D deficient status. Our objectives are to examine the sun exposure behaviours of different United Kingdom sectors and to identify if their exposure is enough to maintain winter circulating 25-hydroxyvitamin D above deficiency (>25 nmol/L). Data are from a series of human studies involving >500 volunteers and performed using the same protocols in Greater Manchester, UK (53.5° N) in healthy white Caucasian adolescents and working-age adults (skin type I–IV), healthy South Asian working-age adults (skin type V), and adults with photodermatoses (skin conditions caused or aggravated by cutaneous sun exposure). Long-term monitoring of the spectral ambient UVR of the Manchester metropolitan area facilitates data interpretation. The healthy white populations are exposed to 3% ambient UVR, contrasting with ~1% in South Asians. South Asians and those with photodermatoses wear clothing exposing smaller skin surface area, and South Asians have the lowest oral vitamin D intake of all groups. Sun exposure levels prevent winter vitamin D deficiency in 95% of healthy white adults and 83% of adolescents, while 32% of the photodermatoses group and >90% of the healthy South Asians were deficient. The latter require increased oral vitamin D, whilst their sun exposure provides a tangible contribution and might convey other health benefits.

## 1. Introduction

The health benefits of vitamin D for the musculoskeletal system are established [1], and vitamin D has also been implicated in the prevention of a range of other diseases [2,3], although there is less clarity of cause and effect for conditions other than bone health [4]. The body normally obtains vitamin D both orally (diet plus/minus supplementation) and by cutaneous synthesis upon skin exposure to ultraviolet radiation (UVR), specifically the UVB (280–315 nm) component [4]. Cutaneous synthesis is believed to be the major source for most people even though sun exposure can be limited by climate and culture. In the United Kingdom (UK) and similar latitudes there is the issue of seasonal variation in the availability of UVB, with very little cutaneous synthesis occurring from the end of September to early March (autumn and winter in the Northern Hemisphere) [5,6]. The situation is made more complex by a wide range of societal changes over recent decades, with potentially reduced skin exposure in daily life, but increased vacation time and vacations in sun-rich environments [7]. This is a cause for concern and a reason to ask whether the population receives enough sun exposure to serve its vitamin D needs.

These concerns are at odds with a major public health drive of the past several decades to reduce sun exposure because of increases in skin cancer [8]. This has led to widespread advice to reduce sun exposure at times when UVB is highest and also to the encouragement of the use of sun protective measures when outside [8]. Finding the balance between enough sun exposure for vitamin D synthesis but not too much to significantly increase the risk of skin cancer is complex, not least because there is no universally accepted value for a target level of circulating 25-hydroxyvitamin D (25(OH)D), the measure of vitamin D status. For this work, we considered a 25(OH)D concentration <25 nmol/L as deficient (following SACN, [4]) and <50 nmol/L as insufficient (following the IOM and EFSA, [9,10]). Using the same definitions, data modelling research reveals that it is theoretically possible to avoid vitamin D deficiency year-round in the UK without incurring skin erythema (a proxy for heightened risk of skin cancer) at least for the white Caucasian population [11,12]. At middle to high latitudes, a seasonal cycle in circulating 25(OH)D was observed, lagging slightly behind the seasonal cycle in solar elevation [13]; consequently, avoiding deficiency throughout winter required higher levels of 25(OH)D to be attained by the end of summer.

Here, our objectives were to examine the sun exposure habits of different UK population sectors and identify if their sun exposure year-round is enough to maintain winter vitamin D status above the level of deficiency (25(OH)D > 25 nmol/L). The data come from a series of both intervention and longitudinal observational studies of the vitamin D status [14,15,16,17,18,19] of community-dwelling volunteers, supported by the long-term monitoring of the spectral UVR representative of the Manchester (53.5° N) metropolitan area [20]. The latter provided the ambient UVR levels for data interpretation and allowed us to account for inter-annual changes in incident UVR due to changing weather from year to year. The same protocols were maintained across all studies, with consistent data collection and analysis.

## 2. Materials and Methods

### 2.1. Studies and Population Groups

The human in vivo data used in this work were obtained from a series of studies which took place over the 5-year period of 2007–2011 and involved 578 volunteers [14,15,16,17,18,19] in Greater Manchester, UK (53.5° N). All studies received ethical and institutional approval and are described in detail in the associated references. Common methods for the data used here are outlined below. The population groups studied were healthy adolescents (12–15 year), working age adults (20–60 year), and people with photodermatoses (skin disorders caused or aggravated by sun exposure). For healthy working adults, two different groups, white Caucasians (skin type I–IV) [14,15] and South Asians (skin type V) [16,18], were involved, while adolescents were white Caucasian [19]. The final group were photosensitive adults of mixed skin type and wider age range, although predominantly white Caucasian and <60 year [17].

The sun exposure data are from observational studies [15,16,17,19] that followed the relevant group over a period of 12 months, covering the maximum and minimum of annual variation in both sun exposure and circulating 25(OH)D. The data recording weeks (1 or 2 per season) were chosen to be representative of the different seasons of the year. While exact weeks varied by study (e.g., to account for the school year), the weeks for all studies fell in winter (January), spring (March/April), summer (June/July) and autumn (September/October). Age- and skin type-matched winter baseline 25(OH)D data from UVR-intervention studies were used to support winter 25(OH)D data from the observation studies: study performance in different years allowed some measure of year-to-year variation in vitamin D status.

### 2.2. Personal Sun Exposure Assessments

During data recording weeks, volunteers wore 2 polysulphone dosimeter badges [21], one badge from Monday to Friday and a second on weekend days, to record personal UVR exposure as the total erythema effective dose expressed in SED (Standard Erythema Dose), where 1 SED = 100 J/m^2^ erythema effective UVR [22]. Volunteers also completed daily sun exposure diaries [15,16,17,19] which recorded a range of data including clothing worn (by selecting from a series of examples), sun protection used, and activities, as well as a binary indicator (inside/outside) for each 15 min period of the day. This covered the hours 06:00 to 20:00; thus, if the dog were taken for a walk between 13:05 and 13:40, boxes 13:00–13:15, 13:15–13:30, and 13:30–13:45 would be marked. In assessing the time outside, each positive (outside) 15 min time block was assigned as a 10 min exposure since isolated time blocks could in reality represent anything from a couple of minutes to 15 min, yet several contiguous time blocks would likely have at least one full 15 min period included [15,16,17]. Ten minutes was identified as a sensible average exposure for a time block. Positive time blocks were summed for each day and each participant, from which the median for each population group and each season was calculated. Data from Reference [19] has been analysed here in the same way for consistency (in the original publication, the full 15 min was assigned to each positive time block). Information on clothing worn was used to estimate the skin surface area exposed using the rule of nines. Any other relevant activities, such as holidays, were also recorded by interview with researchers, including at attendance for blood sampling, which was also performed seasonally.

### 2.3. Dietary Vitamin D and Vitamin D Status

During the same weeks as personal sun exposure assessments, volunteers completed vitamin D dietary logs to record daily intake of vitamin D-containing foods and supplements. A blood sample was also taken to determine the level of circulating 25(OH)D as the biological outcome of sun exposure. 25(OH)D was measured using high-performance liquid chromatography as described in Reference [23].

### 2.4. Ambient UVR Monitoring

Erythemal irradiance has been measured at the Manchester, UK site (Latitude 53.47° N, Longitude 2.3° W, altitude 76 m) using broadband (Kipp and Zonen UVS erythemal UV meter) and spectral instruments (Mark III Brewer spectrophotometer and Biospherical GUV multifilter radiometer) since the late 1990s [20]. The broadband and multifilter instruments record continuously, producing 1 min averages to provide good temporal coverage. All studies were conducted in the Greater Manchester area, so the measured data provide representative ambient UVR (on a horizontal surface) available to the volunteers. We compared the ambient UVR (total for the entire 5 or 2 days that a badge was worn) to the badge dose, bearing in mind that the badge was worn on the lapel to better represent the non-horizontal surfaces of the human body [24]. In addition, the city location reduces street-level UVR due to buildings blocking radiation from both sun and sky [25], while the ambient UVR comes from monitoring instruments with a full hemispherical field of view. Our long-term monitoring of UVR allowed sun exposure to be put in context of both the actual solar radiation available during the study days and the typical exposure that might have resulted from the same behaviour in average or climatological conditions or at different locations (from detailed climatological modelling of the region validated against the measurements) [11].

### 2.5. Data Analyses

Post hoc comparisons were made between cohorts using the Mann-Whitney test, and within-season weekday vs. weekend by the Wilcoxon signed-rank test, without adjustment for multiple testing. Analyses were performed using GraphPad Prism v7 (GraphPad Software, La Jolla, CA, USA).

## 3. Results

### 3.1. Volunteer Demographics

Population group demographics of volunteers completing each study are presented in Table 1.

### 3.2. Daily Time Spent Outdoors

Median time spent outdoors for each population group is shown in Table 2. Spring and summer data are shown, as these are the seasons when there is sufficient UVB to initiate significant cutaneous production of vitamin D: from approximately October to March in Manchester, UK, solar UVB is ineffective for vitamin D synthesis [15]. For weekdays, there was a consistent time outdoors in spring and summer for all groups, as might be expected from structured work and school days. The most noticeable difference in median time outdoors between weekdays and weekend days was seen for the white adults, who spent significantly longer outdoors at the weekend in both seasons (*p* < 0.01). Adolescents also spent significantly longer outdoors on weekends than weekdays in summer (*p* < 0.05). There was no appreciable change in time outdoors from weekday to weekend in the photosensitive or South Asian groups. Across all groups, and particularly for the adolescent group, the larger interquartile ranges for time outdoors at the weekend showed the variety of behaviours during free time. The weather impact was evident in the white adult weekend times, as the recording weeks in that particular year provided for an unusually warm and sunny April weekend and, by contrast, an inclement July weekend. This was reflected in the time outdoors, with 95 min in April and 70 min in July. Time outdoors for photosensitive volunteers was not particularly low, but it did not vary between weekday and weekends, suggesting that, unlike non-photosensitive adults, they do not increase their sun exposure time when unrestrained by work. Time outdoors for the South Asian (skin type V) adults also showed no consistent increase from weekdays to weekends, and in this case, it was not affected by distinctive weekend weather.

### 3.3. Skin Surface Area Exposed and Use of Sun Protection

From the sun exposure diaries, we obtained information on the clothing worn by the volunteers during the time they were outside, and we used this to estimate the percentage of skin surface area exposed for each person on each day. This is summarised in Table 3 by population group.

As expected from comfort considerations, volunteers in all groups wore most clothing in the winter, with often just hands and face exposed. Photosensitive volunteers generally exposed the lowest skin area in other seasons and were most consistent as a group (smallest interquartile range). They retained a dress style that exposed only hands and face throughout the year, whereas healthy working adults and adolescents all had modest increases in skin area exposed during the warmer spring and summer months, both during the week and on weekends. For those spring and summer months, the difference between the weekday and weekend skin area exposed was small, with consistently a median of 3% higher on weekends for white adults and no consistent difference for other groups.

Photosensitive volunteers, as would be anticipated, used the most sun protection, with over 60% of participants recording sun protection use at least once in their exposure diaries. The next largest users of sun protection were the white adults, 31% recording use of some form of sun protection in the summer, whilst no more than 10% of South Asian adults used sun protection factor (SPF)-containing products in any season. In the adolescent study, no sunscreen use was recorded, though a small number occasionally used SPF-containing cosmetic products.

### 3.4. Personal Sun Exposure Measurements

**Figure 1:**
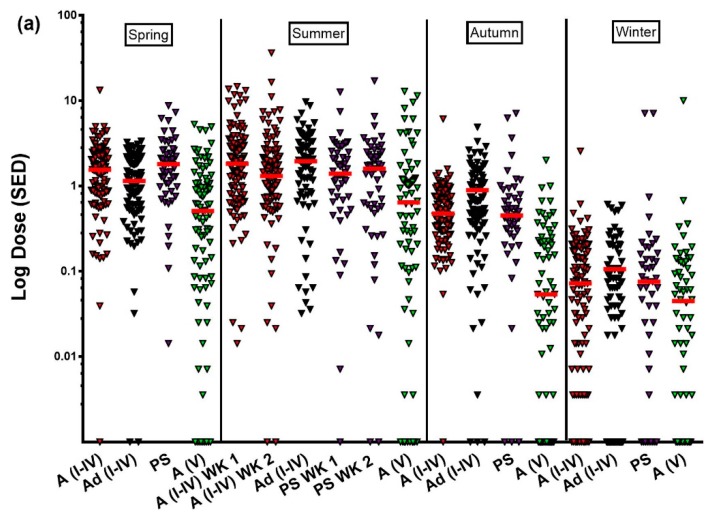

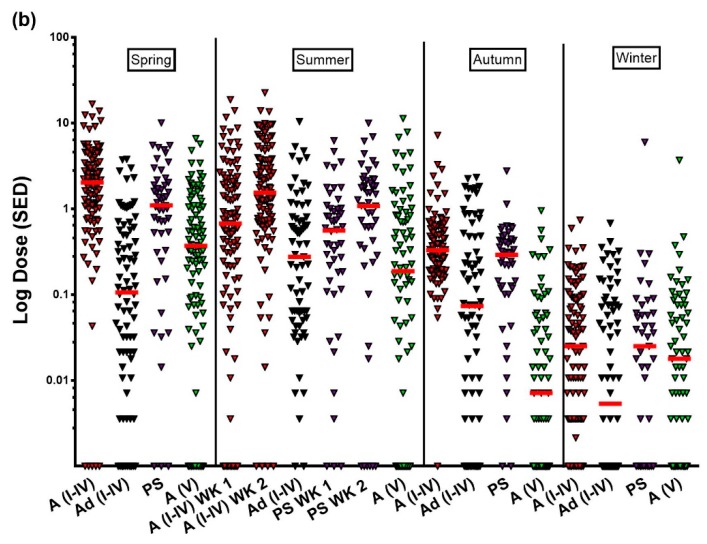
Dose of UVR received (SED) as measured by polysulphone dosimeter badges worn by volunteers: (**a**) Weekday data; (**b**) Weekend data. Red bars show the median for the group. A: adult; Ad: adolescent; PS: photosensitive; WK: week; I–IV and V refer to volunteer skin types.

Data for personal sun exposure, measured using polysulphone badges, are shown in Figure 1, arranged by season and population group. The white adult and photosensitive groups wore badges for two consecutive weeks in their summer seasons (while the other groups wore badges for one week), and each week is represented independently in the figure to show week to week variation.

The UVR doses (SED) derived from the dosimeter badges illustrated the natural variation of UVR from the summer peak to the winter trough, modified by personal behaviour (note the logarithmic scale for dose). The South Asian adult (skin type V) volunteers showed distinctly lower recorded doses than all other groups during weekdays, and they were lower than other adult groups on weekends. This was at odds with the exposure times from the diaries, where the South Asian adult times spent outdoors were commensurate with the photosensitive group and higher than the white adults except for the spring weekend. For the adolescent study, the weekday badge doses followed the expected annual pattern in the same way as the adult badge doses. Weekend adolescent doses were lower and had a wider range than the weekday doses, the latter being controlled to a large extent by the school day. We also noted a large number of zero exposures on weekends, some of which may be attributed, anecdotally, to lack of compliance (not wearing the dosimeter badge), or they might reflect that, despite recording being “outside” in sun exposure diaries (Table 2), the volunteers were actually in shaded or covered places such as shopping centres. The doses shown in Figure 1 are those from individual badges; therefore, the weekday badge represents total exposure over 5 days, and the weekend badge represents the total over 2 days. Despite being only two days, the weekend doses recorded in the adult studies were broadly similar to the weekday doses, meaning that the two day weekend was respondible for approximately half the overall adult exposure to UVR. In contrast, as a cohort, children gained the majority of their UVR exposure during the school week, with weekend contributions varying greatly from person to person.

A small number of participants took holidays during recording weeks, and their data represents sun exposure at locations other than Manchester, UK. These were retained in the analysis as representative of population lifestyle, and they are identified most easily in the winter data as occasional uncharacteristically high exposures for the climate and time of year.

### 3.5. Ambient UVR Monitoring

Median badge exposure as a proportion of the horizontal ambient UVR was close to 3% for healthy white adult skin type I–IV volunteers, but it was lower, at approximately 1%, for South Asian (skin type V) volunteers. For the white adolescents, it was <3% for weekdays but <1% on weekends. The photosensitive adults had median badge exposures that were similar to the healthy white adults for a study conducted in the same year (same ambient UVR), except for the spring and summer weekends when, as previously noted, they did not increase time outdoors in the same way as the healthy adults. Nonetheless, median weekend exposures still exceeded those for both white adolescents and South Asian adults.

Furthermore, data from this monitoring allowed us both to rule out large changes in ambient UVR as a cause of differences in results between studies, and to explain some of the less intuitive results (e.g., white adult weekend exposures as discussed under Table 2).

### 3.6. Dietary Vitamin D Intake

**Figure 2:**
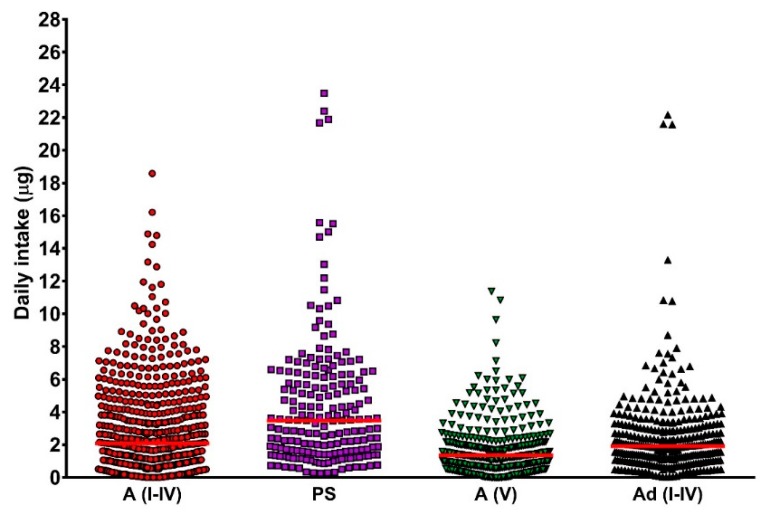
Daily oral vitamin D intake for all volunteers (all seasons). There were no significant differences in intake between seasons [15,16,17,19]. Red bars are the median for each group. A: adult; Ad: adolescent; PS: photosensitive; I–IV and V refer to volunteer skin types.

Data from weekly diet logs, completed in all studies included here, showed that levels of oral vitamin D intake were low (Figure 2) and there was not any significant seasonal variation [15,16,17,19]. Median intake was less than 4 µg per day (160 IU per day) in all groups, with the lowest median intake among the South Asian adults (1.32 µg per day) [16]. Use of supplements was limited to a very small number of volunteers amongst the white adolescents and South Asian adults. Amongst the white adults, approximately 26% were taking supplements, and this increased to almost 35% in the photosensitive group. Median oral intakes were well below the value of 10 µg per day recommended by SACN as a daily oral vitamin D intake for all sectors of the UK population (other than infants <1 year of age, for whom 8.5 µg per day is recommended) [4], that being the dietary level intended to prevent deficiency throughout the year.

### 3.7. Assessment of Winter 25(OH)D Levels

**Figure 3:**
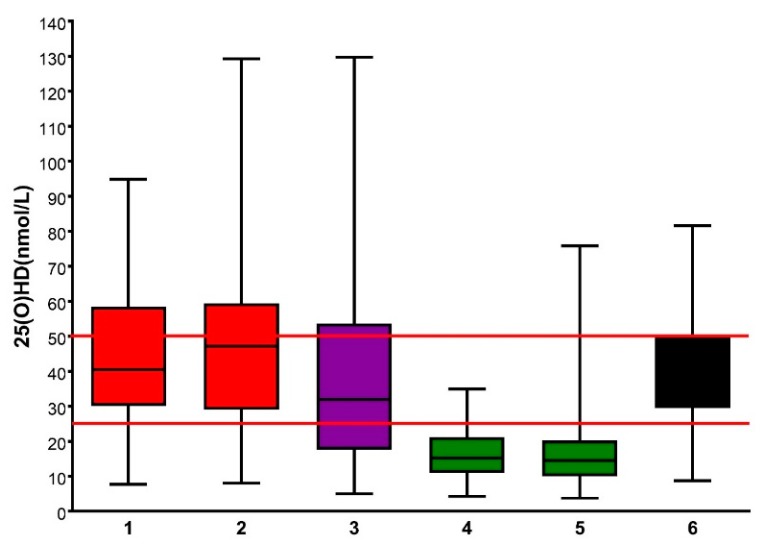
Winter serum 25(OH)D measurements, by cohort. The box and whisker plots show the median (black line), interquartile range (box), and extreme range (whiskers). The red horizontal lines show levels of deficiency (<25 nmol/L) and insufficiency (<50 nmol/L). Key to the groups: 1: white adult intervention, 2007; 2: white adult observation, 2008; 3: photosensitive adults, 2008; 4: S. Asian adult intervention, 2009; 5: S. Asian adult observation, 2010; 6: white adolescent, 2011. Data are from References [14,15,16,17,18,19].

Given the variability in sun exposure, and hence vitamin D status, throughout the year, we recalled the suggested public health goal of SACN [4], that the population as a whole should remain above deficiency status (25(OH)D > 25 nmol/L) throughout the year, and we inspected the winter 25(OH)D status for all volunteers from both observational studies and (pre-exposure) intervention studies (Figure 3). While there was some variation between volunteer groups from different years and studies, the vast majority (95%) of healthy white adults avoided winter-time deficiency, while this was less so in white adolescents (83%). The photosensitive group (who were mostly skin type I–IV) showed a substantial proportion (32%) in the deficiency range. By contrast, >90% of all South Asian (skin type V) healthy volunteers were deficient during the winter. For the population with white skin, the majority had a vitamin D status classified as “insufficient” (25(OH)D < 50 nmol/L) during winter, while those with the natural pigmentation of skin type V rarely reached above “deficient” status.

## 4. Discussion

The results from this range of studies in the North of England, UK showed that there were distinct differences in the study groups in terms of sun exposure behaviour and vitamin D outcomes. The different winter 25(OH)D levels reflected the influence of several factors. Firstly, there were intrinsic factors that the volunteer could not change, most notably skin colour. Then there were behavioural differences (both chosen and imposed through medical conditions) such as time and activity outside, clothing worn, photoprotective measures, and diet; all can potentially be modified in healthy people, but they may also be subject to societal and cultural restrictions.

Oral intake of vitamin D was consistently low throughout the year for all groups, most particularly for South Asians, and sun exposure was therefore confirmed as a major source of vitamin D. An annual cycle in vitamin D status has been previously observed [15,16,17,19], albeit with a small amplitude for those with skin type V [16].

Whether or not sun exposure is enough (from a vitamin D perspective) depends on how the target vitamin D status is defined and whether supplement use or food fortification is included. In principal, strict avoidance of sun exposure (for the reduction of any sunburn erythema or skin cancer risk) could be compensated by vitamin D supplementation at a sufficient level. SACN recommends an oral vitamin D intake of 10 µg per day year-round for the majority of the population, which in practice currently implies supplement use for most people [4]. Clearly, this was not observed amongst the sampled populations of Greater Manchester. Practically, the white population obtained most of their vitamin D through sun exposure.

The target for vitamin D status has been set at the lowest bar for avoiding deficiency, assessed as having 25(OH)D > 25 nmol/L at the winter sampling point, since winter is the time of lowest vitamin D status. For all healthy white groups, their sun exposure throughout the year was enough to keep the great majority above vitamin D deficiency status in winter, although personal variation in behaviour was such that too many individuals became deficient by the norms of population nutrition (i.e., where 97.5% of the population should be within the desired range, in this case 25(OH)D > 25 nmol/L). However, it has been shown that for the white population in the UK, vitamin D deficiency can be theoretically avoided by 97.5% of the population if 9 min of noon-time sun exposure is achieved daily from March to September [12]. Our volunteers clearly exceeded this time period (Table 1) in the recording weeks; while time outdoors did not always occur during the midday hours, it happened at other times of day when the ambient UVR was less because the solar elevation was lower. However, the work by Webb et al. [12] also assumed that 35% of skin area was exposed during the 9 min, at least in the warmer summer months of June to August. It is clear that the majority of our volunteers did not, in general, expose anything approaching this amount of skin, even in summer. Nonetheless, the sun exposure behaviour of much of the population is enough to provide for vitamin D needs, defined as avoiding winter deficiency. The efficacy of vitamin D synthesis in unclothed skin may be modified if the UVR is blocked or partially blocked by sun cream products and cosmetic products that provide an SPF. However, there is no evidence that vitamin D synthesis is entirely prevented by use of sun protection products [26], and such products are considerably less effective in practice than advertised, because they are not usually applied at the concentrations used in standard SPF testing [27]. Thus, while skin area exposed could be modified by use of SPF-containing products, the adequacy of application by our participants was not known, and we only recorded the use of sun protection.

The situation for the South Asian (skin type V) population is different. The theoretically required noontime exposure for this skin type has been estimated as 25 min [28], with the same requirements of 35% skin area exposed in June–August. Again, the time outdoors achieved by our adult participants appears to reach this target, albeit not necessarily during the noon hours, but sun avoidance techniques and clothing reduce both the actual UVR exposure and the skin area exposed. The result is evident in the vitamin D status of this population, who are predominantly deficient in the wintertime and, indeed, all year-round [16]. The extra sun exposure required as a result of constitutive skin pigmentation [29] is both demanding and at odds with the cultural practices of many. Observation, discussion, and focus group work [30] lead to the hypothesis that adults of South Asian origin can be culturally inclined to seek shade from the sun and value pale skin, while the white community are inclined to seek out the sun and a tan. Thus, the South Asian group may be seeking shaded locations outdoors, which explains why the two groups have a different correlation between time outside (recorded in diaries) and UVR exposure (SED recorded by badges). Given the very low 25(OH)D levels of the South Asian population, sun exposure is not enough, and alternative sources of vitamin D should be considered.

Clearly, patients with photodermatoses (a range of skin disorders triggered by abnormal sensitivity to UV and/or visible radiation, overall affecting substantial numbers of people) face unique challenges with respect to sun exposure. They employ, and are medically advised to use, behaviours and measures to minimise sunlight precipitation or aggravation of their disorder. This was evident in the photosensitive group and reflected in a substantial proportion being deficient in winter. Moreover, deficient and insufficient statuses frequently persisted even at summer-end, with potentially greater risk to musculoskeletal health [17], and oral supplementation is advised.

Solar UVR exposure may have other health benefits than vitamin D synthesis. Recently, studies in humans in vivo showed that exposure to UVR (5% UVB, 95% UVA) modulated the cutaneous endocannabinoid system [31], while exposure to UVA radiation (315–400 nm) reduced blood pressure [32]. Further studies are required to assess the impact of sun exposure on these and further potential health benefits beyond vitamin D synthesis, while the latter is the current established benefit and hence the focus of this work.

## 5. Conclusions

The results from a series of studies show that sun exposure in an urban/suburban environment in northern England, UK was low (<3% ambient UVR) across different age groups and ethnicities, but it was particularly low (~1%) amongst South Asians. Nonetheless, this exposure was a major source of vitamin D, since dietary intake and supplement use was shown to be low. The winter-time vitamin D status of these community-dwelling volunteers has been taken as an indicator of whether they received “enough” UVR exposure during the year (principally March–September, and on sunny holidays). Sustaining 25(OH)D > 25 nmol/L has been defined as a marker of adequate sun exposure for vitamin D purposes. This was achieved in real-life by 95% and 83% of white skinned healthy adults and adolescents, respectively, although an equivalent nutritional target would be 97.5% of the population. However, South Asians (skin type V) faced a combination of factors adversely affecting vitamin D status (less sun exposure of the skin when outdoors, less oral vitamin D, and melanisation), and sun exposure did not provide adequately for them, at least from a vitamin D perspective (>90% deficient in winter). This population group, and groups with medical reasons to minimise sun exposure (as in the photodermatoses), require increased oral vitamin D, whilst the sun exposure they gain provides a tangible contribution and could provide other health benefits.

## Figures and Tables

**Table 1:** Volunteer demographics.

Population Group	Total *n*	Sex (*n*)	Skin Type (*n*)
White adults	218	59 M; 159 F	I = 23, II = 116, III = 72, IV = 7
White adolescents	131	51 M; 80 F	I = 15, II = 38, III = 62, IV = 16
Photosensitive adults	53	9 M; 44 F	I = 11, II = 15, III = 13, IV = 7, V = 4, VI = 3
S. Asian adults	176	123 M; 53 F	V = 176

**Table 2:** Median (IQR) minutes spent outdoors per day.

Season	White Adults (Skin Type I–IV)	White Adolescents (Skin Type I–IV)	Photosensitive Adults	S. Asian Adults (Skin Type V)
Spring weekday	52 (34–78)	62 (40–105)	70 (46–103)	72 (46–104)
Summer weekday	51 (34–82)	76 (43–110)	75 (45–112)	63 (34–110)
Spring weekend	95 (60–150)	73 (36–122)	80 (35–118)	60 (30–90)
Summer weekend	70 (30–100)	80 (36–131)	75 (40–125)	80 (38–130)

**Table 3:** Percentage of skin surface area exposed during study weeks.

Day	Population Group	Median (IQR) % Skin Surface Area Exposed			
		Spring	Summer	Autumn	Winter
Weekday	White adults (skin type I–IV)	11 (8–13)	14 (11–19)	8 (8–16)	7 (7–15)
	White adolescents (skin type I–IV)	11 (8–17)	14 (8–17)	8 (8–14)	8 (8*–*14)
	Photosensitive adults	11 (11–13)	11 (11–17)	11 (11–11)	11 (11–11)
	S. Asian adults (skin type V)	12 (9–17)	14 (10–18)	10 (8–15)	8 (8–11)
Weekend	White adults (skin type I–IV)	14 (11–19)	17 (14–26)	8 (8–28)	8 (8–14)
	White adolescents (skin type I–IV)	13 (8–19)	14 (11–19)	14 (8*–*17)	8 (8*–*16)
	Photosensitive adults	14 (11–17)	11 (11–17)	11 (11–11)	11 (11–11)
	S. Asian adults (skin type V)	10 (8–16)	14 (8–18)	8 (8–14)	8 (8–10)
